# GABA_B_ receptor modulation during drug-memory reconsolidation: current evidence and translational priorities for relapse prevention

**DOI:** 10.3389/fphar.2026.1944369

**Published:** 2026-09-23

**Authors:** Kuisheng Sun, Li Deng, Tao Li, Wenhua Huang, Wanli Lin

**Affiliations:** 1 Gaozhou People’s Hospital, Gaozhou, Guangdong, China; 2 Guangdong Provincial Key Laboratory of Medical Biomechanics, Department of Human Anatomy, Guangdong Engineering Research Center for Translation of Medical 3D Printing Application, School of Basic Medical Sciences, Southern Medical University, Guangzhou, Guangdong, China

**Keywords:** baclofen, drug memory, GABA_B_ receptor, memory reconsolidation, neuropharmacology, pharmacokinetics/pharmacodynamics, positive allosteric modulator, relapse prevention

## Abstract

Relapse after abstinence is sustained in part by drug-associated memories reactivated by cues, contexts, actions, and interoceptive states. Reconsolidation-based interventions seek to modify a retrieved memory during transient lability. GABA_B_ receptors suppress transmitter release, hyperpolarize neurons, and inhibit adenylyl-cyclase-cAMP-protein-kinase-A signaling, providing biological plausibility for temporally aligned modulation. This structured narrative review organizes the literature using a non-comparative evidence-domain framework. Direct retrieval-window evidence remains limited to two local baclofen studies in male mice using morphine conditioned place preference; both reported retrieval dependence, but only the agranular insular cortex study included a delayed-treatment comparison. Earlier systemic baclofen studies conducted during repeated extinction and post-conditioning GABA_B_ positive allosteric modulator studies support memory-related pharmacology but do not isolate a retrieval window. A systemic post-reactivation baclofen study extends the evidence beyond local microinjection, whereas human baclofen and ASP8062 studies define tolerability, exposure, CNS pharmacodynamics, and non-timed clinical boundaries rather than reconsolidation efficacy. Translation requires a validated reactivation procedure, adequate pharmacokinetic coverage and CNS pharmacodynamic engagement, randomized retrieval and timing controls, post-acute persistence, and protection against sedation, task disengagement, and repeated-cue confounding. The evidence supports a falsifiable development hypothesis, not memory erasure or a universal anti-addiction therapy.

## 1 Introduction

### 1.1 Addiction relapse as a pathological memory problem

Relapse after abstinence reflects more than acute withdrawal or short-term failure to stop drug use. Repeated pairing of drug effects with external cues, contexts, actions, and interoceptive states creates durable drug-reward or drug-relief memories that can later evoke craving and renewed seeking. These memories interact with habit formation, negative affect, and weakened executive control across the addiction cycle; they are therefore one component of a multi-process relapse syndrome rather than a complete explanation of substance use disorders (Everitt, 2014; Volkow and Boyle, 2018).

Conventional cue exposure mainly establishes new inhibitory learning and does not necessarily eliminate the original cue-drug association. Drug seeking can therefore re-emerge through spontaneous recovery, renewal, drug priming, or stress. A memory-focused relapse strategy must address not only whether a conditioned response is suppressed during treatment, but also whether the underlying association was retrieved, destabilized, updated, and durably restabilized (Kuijer et al., 2020; Chen et al., 2021). In this review, GABA_B_ receptor modulation is evaluated as a retrieval-window intervention rather than as a general maintenance medication.

### 1.2 Reconsolidation as a clinically relevant intervention window

A consolidated memory can become transiently labile after retrieval and may require new protein synthesis and synaptic reorganization to restabilize (Nader et al., 2000). This principle has been extended to addiction memories: retrieval-dependent interference can reduce later cocaine seeking in animals, and retrieval followed by extinction has reduced laboratory cue reactivity in abstinent heroin users (Lee et al., 2005; Xue et al., 2012). In the clinical experiment reported by Xue et al. (2012), the principal human outcome was cue-elicited craving rather than objectively verified real-world relapse; the study therefore supports memory updating under controlled conditions but does not by itself establish relapse prevention.

Conditioned-stimulus (CS) retrieval-extinction uses brief re-exposure to a learned cue or context before extinction training. Its rationale is that a limited mismatch between expectation and outcome can open a reconsolidation window, whereas very weak retrieval may leave the memory stable and prolonged nonreinforcement may instead engage extinction. In cocaine models, extinction delivered within the putative window can attenuate later memory expression, but effects vary with retrieval duration, prediction error, memory strength, and relapse test (Sartor and Aston-Jones, 2014; Exton-McGuinness et al., 2015; Kuijer et al., 2020).

Unconditioned-stimulus (UCS) retrieval aims to reactivate a shared representation of the drug outcome rather than one specific environmental cue. In preclinical work, UCS retrieval followed by extinction reduced relapse-like behavior after prolonged abstinence and involved basolateral-amygdala AMPA-receptor endocytosis (Luo et al., 2015). CS and UCS procedures should therefore be treated as distinct opening operations. UCS retrieval may broaden memory coverage, but using a drug or drug-like stimulus to retrieve the representation creates ethical and re-reinforcement concerns for human translation.

Retrieval is therefore necessary but not sufficient for a reconsolidation claim. A persuasive experiment should specify the target memory, show that treatment depends on retrieval, test the temporal boundary of the post-retrieval effect, demonstrate persistence, and exclude locomotor, cognitive, motivational, or acute expression effects. These criteria are used below to separate demonstrated retrieval-window effects from supportive evidence that answers a different question (Exton-McGuinness et al., 2015; Chen et al., 2021; Taujanskaitė et al., 2021).

### 1.3 Why focus on the GABA_B_ receptor?

GABA_B_ receptors are inhibitory metabotropic receptors with clinically and experimentally usable ligands. Presynaptic receptors inhibit voltage-gated Ca^2+^ channels and transmitter release, while postsynaptic receptors activate G protein-gated inwardly rectifying potassium (GIRK) channels and reduce neuronal excitability. Through Gαi/o, receptor activation also inhibits adenylyl cyclase (AC), cyclic adenosine monophosphate (cAMP), and protein kinase A (PKA) signaling (Pin and Bettler, 2016). These canonical actions can reduce excitatory drive and intracellular signaling during a post-retrieval interval without requiring the claim that every downstream plasticity process is a direct GABA_B_ target.

Baclofen is an available orthosteric agonist, but its clinical record in substance use disorders is heterogeneous and sensitive to population, endpoint, dose, and adverse effects (Kahn et al., 2009; Agabio et al., 2023). Positive allosteric modulators (PAMs) may amplify endogenous GABA responses with less intrinsic agonism, although compounds differ in efficacy, selectivity, brain penetration, and tolerability (Vengeliene et al., 2018; Maccioni and Colombo, 2024; Wydra et al., 2024). The translational question is therefore narrower than whether GABA_B_ pharmacology is generally anti-addictive: can drug exposure and CNS pharmacodynamic engagement be aligned with a validated memory-reactivation window and produce a persistent memory-relevant effect that is not explained by sedation or behavioral suppression?

### 1.4 Review question and contribution

The focal question is which parts of the GABA_B_ literature directly test drug-memory reconsolidation and which instead provide systemic, human, mechanistic, contextual, or safety support. The review accordingly separates evidence domains rather than placing biologically different studies on one linear quality scale. This distinction allows a local retrieval-timed experiment, a human pharmacokinetic study, and a receptor-trafficking study to remain valuable without implying that they make comparable claims.

The intended contribution is a decision framework linking each evidence domain to the next experiment required for progress. Direct evidence identifies what has been demonstrated under retrieval-window controls; mechanistic evidence identifies testable pathways; systemic and human studies define tolerability, exposure, CNS pharmacodynamic effects, endpoint constraints, and clinical boundaries; and translational design identifies the conditions under which the hypothesis could be supported, narrowed, or falsified. The figures summarize canonical synaptic signaling (Figure 1), candidate signaling convergence (Figure 2), non-comparative evidence domains (Figure 3), and the proposed human development workflow (Figure 4).

**FIGURE 1:**
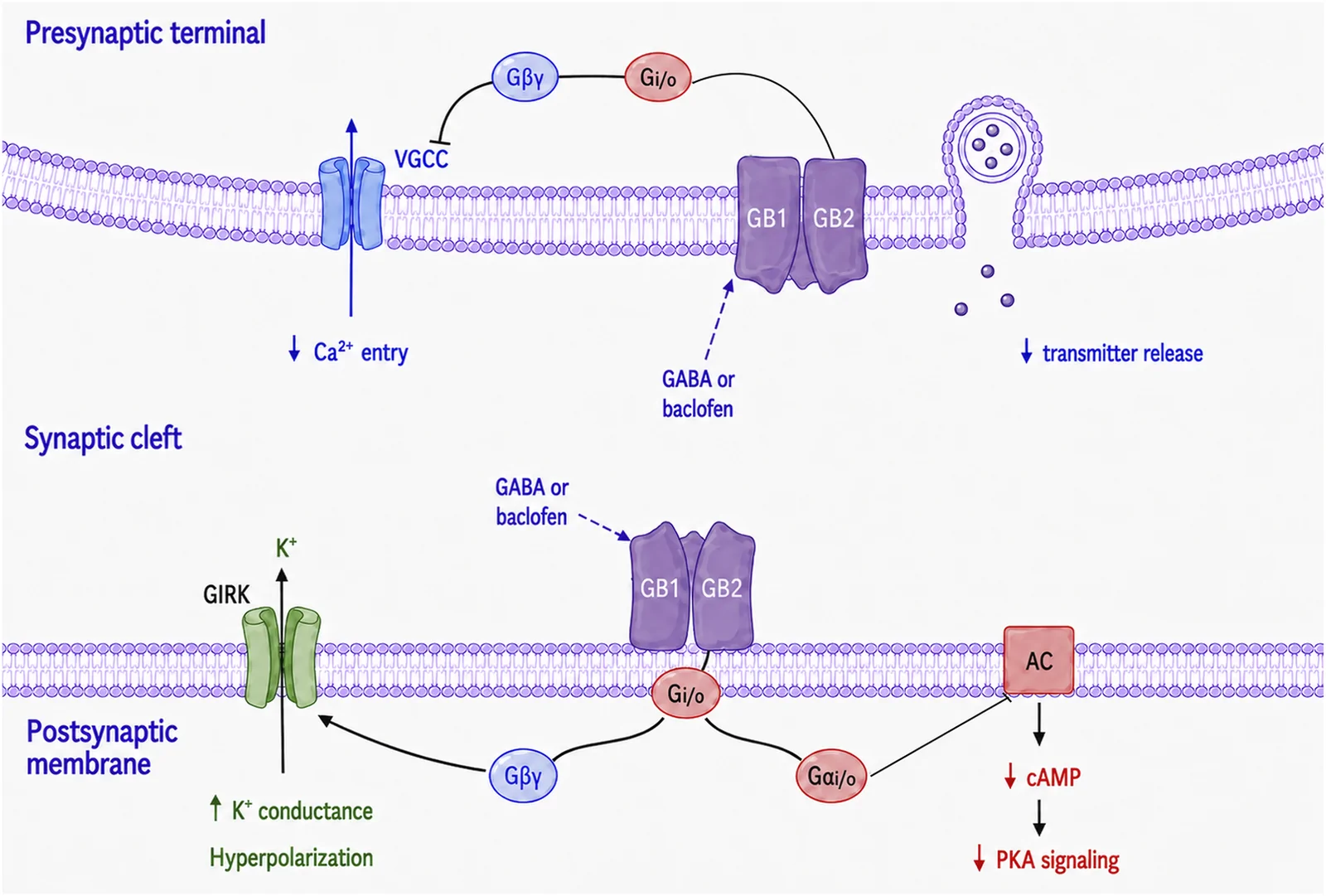
Synaptic localization and canonical signaling of the GABA_B_ receptor. Presynaptic GABA_B_ receptors signal through Gβγ to inhibit voltage-gated Ca^2+^ channels, reducing Ca^2+^ entry and transmitter release. Postsynaptic Gβγ activates GIRK channels, increasing K^+^ conductance and hyperpolarization. In parallel, Gαi/o inhibits AC, lowering cAMP and PKA signaling. GB1 and GB2 are labeled within each receptor to orient orthosteric ligand recognition and G-protein coupling. These are established receptor actions; reconsolidation inference additionally requires retrieval, timing, persistence, and specificity controls.

**FIGURE 2:**
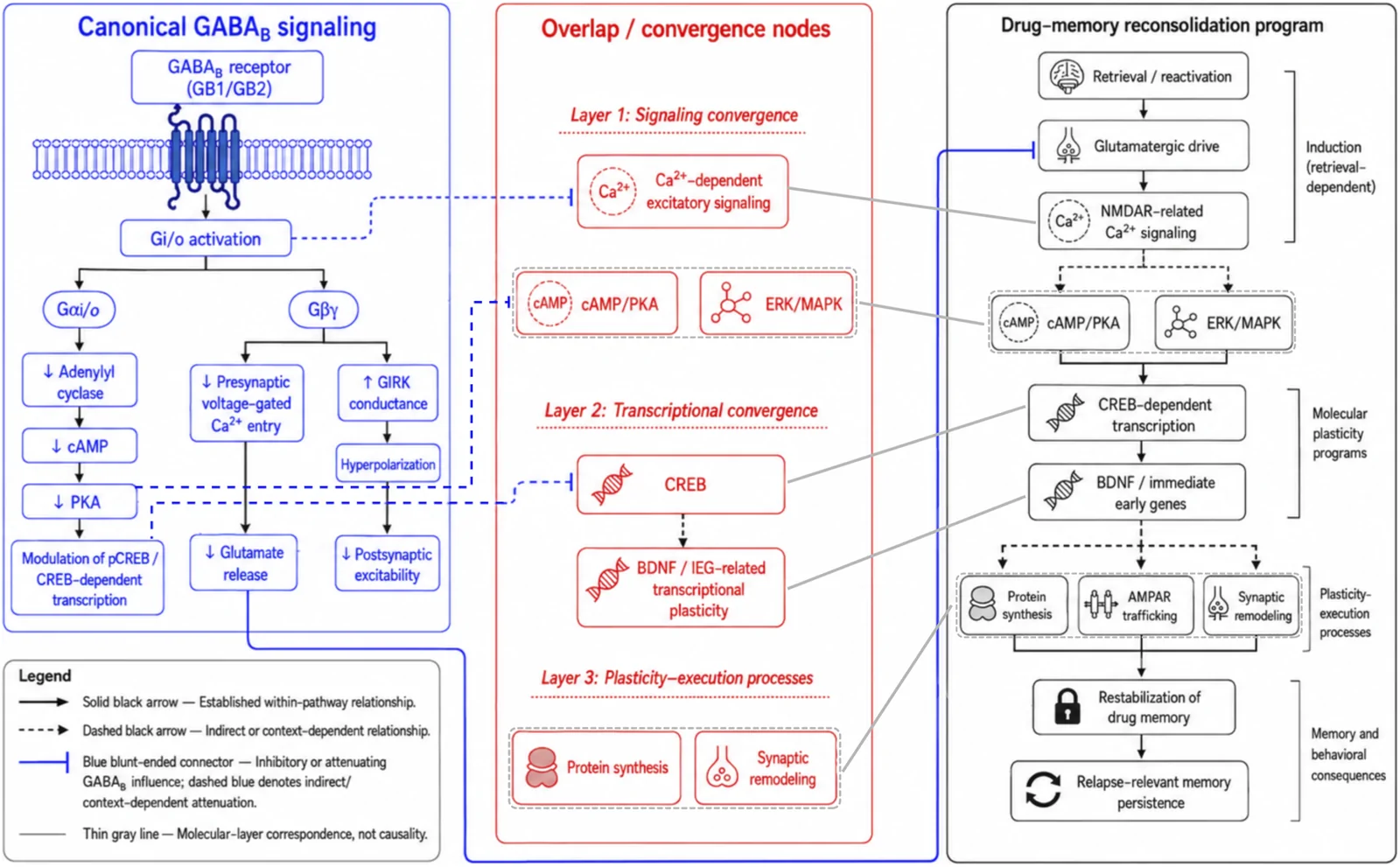
Established GABA_B_ signaling and candidate convergence with memory restabilization. The left panel summarizes canonical receptor actions. The middle panel groups candidate convergence at signaling, transcriptional, and plasticity-execution levels. The right panel schematically summarizes retrieval/reactivation, excitatory signaling, kinase and transcriptional processes, and plasticity-execution events associated with memory restabilization and persistence. The lower-left key defines the connectors: solid black arrows indicate within-pathway relationships; dashed black arrows indicate indirect or context-dependent relationships. Blue blunt-ended connectors indicate inhibitory or attenuating influences, with dashed blue lines denoting indirect or context-dependent attenuation. Thin gray lines indicate molecular-layer correspondence, not causality. The figure does not establish that every connection has been demonstrated in retrieval-timed GABA_B_ experiments; the required experimental controls are described in Section 3.1.

**FIGURE 3:**
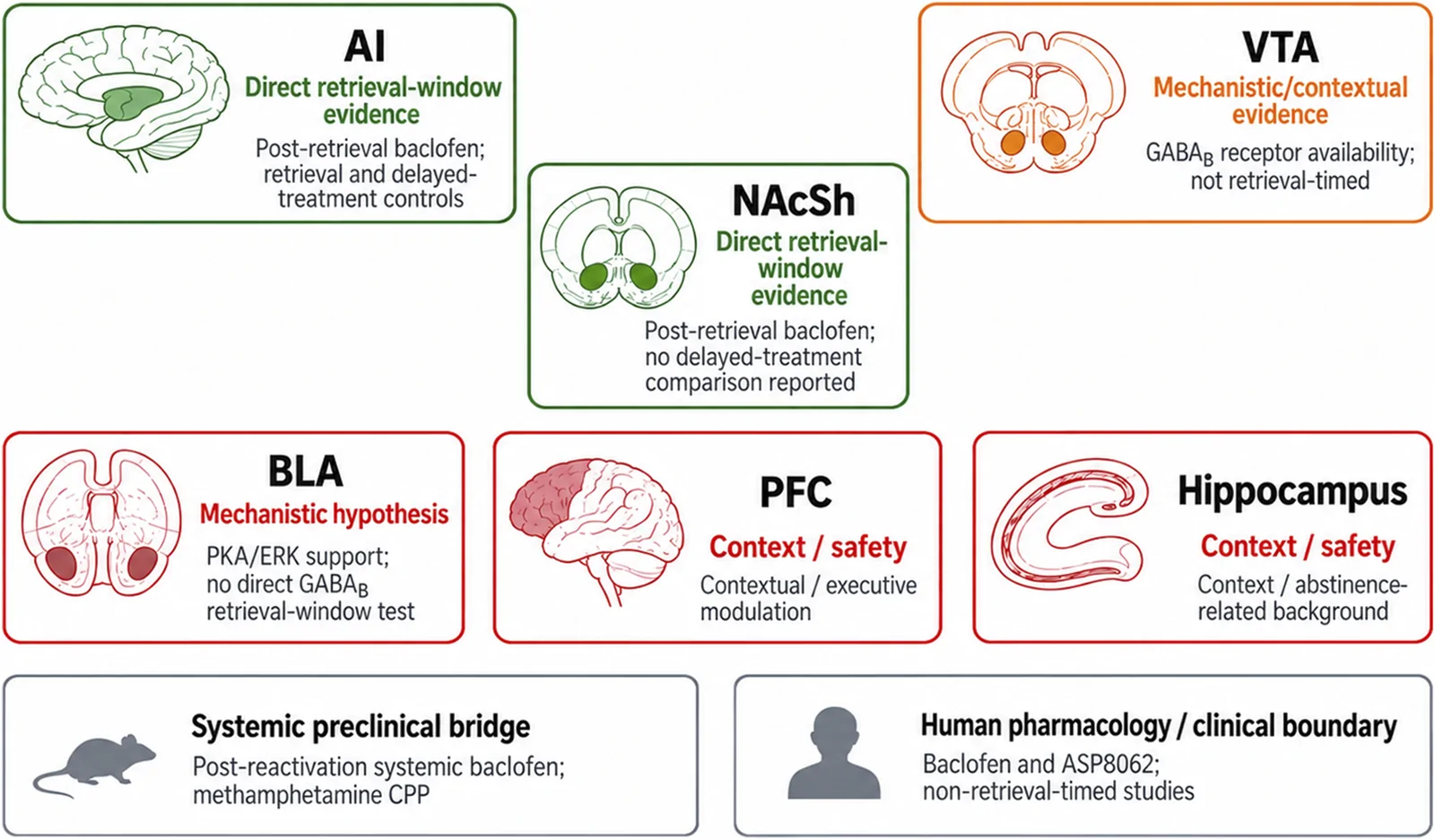
Non-comparative evidence domains relevant to retrieval-timed GABA_B_ modulation. The agranular insular cortex (AI) and nucleus accumbens shell (NAcSh) panels show direct post-retrieval baclofen evidence. The AI study reported retrieval and delayed-treatment controls, whereas no delayed-treatment comparison was reported for the NAcSh study. Ventral tegmental area (VTA) receptor-availability findings provide mechanistic/contextual evidence; basolateral amygdala (BLA) PKA/ERK findings support a mechanistic hypothesis rather than a direct GABA_B_ retrieval-window test. Prefrontal cortex (PFC) and hippocampal findings provide contextual and safety considerations. The lower panels summarize systemic post-reactivation baclofen evidence and non-retrieval-timed human baclofen/ASP8062 studies. Domains indicate the type and inferential relevance of evidence, not a ranking of overall study quality. Brain icons are schematic identifiers, not precise anatomical maps; no interregional causal connections are implied. Study-level controls, shared limitations, and additional extinction/expression-stage contextual evidence are detailed in Section 5 and Table 1.

**FIGURE 4:**
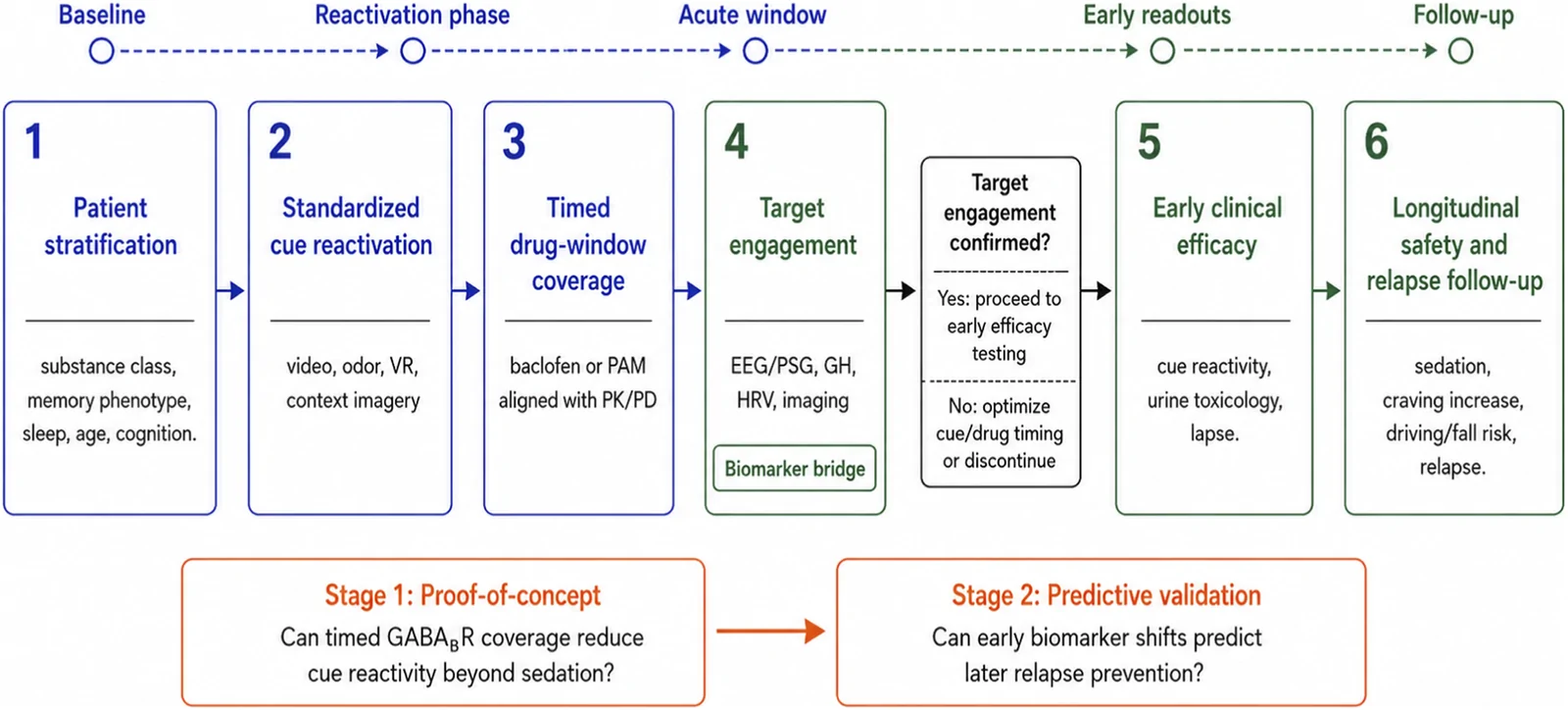
Proposed development workflow for retrieval-timed GABA_B_ intervention. The six steps summarize patient stratification, cue reactivation, timed drug-window coverage, candidate functional/pharmacodynamic readouts, early outcome assessment, and longitudinal safety and relapse follow-up. The broad label “target engagement” and its decision node refer to functional/pharmacodynamic assessment, not proof of receptor occupancy or memory destabilization; individual biomarkers require compound-specific validation and interpretation alongside exposure and specificity controls. “Early clinical efficacy” identifies outcomes proposed for assessment, not established treatment benefit. Stage 1 asks whether timed intervention reduces cue reactivity beyond sedation; Stage 2 proposes testing whether early biomarker changes predict later benefit, rather than reporting completed validation. This is a high-level development overview, not a complete randomized trial protocol. Retrieval and timing comparisons, manipulation checks, post-acute persistence, and equivalent clinical support across arms remain specified in Section 6.4 and Table 2.

## 2 Review approach and evidence-domain framework

### 2.1 Review design and literature identification

This article is a structured narrative review organized using a non-comparative evidence-domain framework; it is not a PRISMA-based systematic or mapping review. Literature identification for the original review involved PubMed, Web of Science, and journal or publisher websites using combinations of terms related to the GABA_B_ receptor, baclofen, PAMs, drug-memory reconsolidation, conditioned place preference, self-administration, relevant brain regions, ASP8062, pharmacokinetics/pharmacodynamics, cue reactivity, and clinical translation. The scope extended from foundational reconsolidation studies published after 2000 to the literature available during manuscript preparation.

During revision, targeted checks were completed on 30 August 2026 for newly added plasticity and contextual-pharmacology references and for the Wang et al. (2020) bibliographic record and English abstract, using PubMed and official journal, publisher, or DOI records. These checks were not treated as a *de novo* systematic search. No pooled effect estimate was calculated because the evidence spans different substances, species, memory paradigms, intervention routes, molecular endpoints, and clinical outcomes. No validated risk-of-bias instrument was applied; the framework below describes inferential relation to the focal retrieval-timed question rather than overall study quality. Because literature identification was narrative rather than systematic, statements about absent evidence are limited to the sources identified and should not be interpreted as proof of exhaustive nonexistence. Generative AI tools were not used to create search records, eligibility decisions, study data, or statistical results.

### 2.2 Descriptive evidence domains and control features

Direct retrieval-window evidence includes studies that administer GABA_B_ modulation after a defined retrieval event and include at least a retrieval/no-retrieval comparison before assessing subsequent memory expression. The relevant control features are reported descriptively rather than converted into a quality grade: retrieval versus no retrieval, immediate versus delayed treatment, persistence after the acute drug state, relapse-like challenge, and behavioral-specificity controls. This approach preserves the factual difference between the two morphine conditioned place preference studies without labeling either study as globally complete or incomplete.

Systemic preclinical bridge evidence tests whether post-reactivation dosing can operate beyond local microinjection but remains limited by species, paradigm, and physiological state. Earlier baclofen studies that embedded treatment within repeated extinction, and post-conditioning PAM studies, are treated as extinction/expression-stage contextual evidence because they do not isolate a retrieval window. Human pharmacology or clinical-boundary evidence addresses tolerability, systemic exposure, CNS pharmacodynamic effects, and patient outcomes; it does not demonstrate reconsolidation unless treatment is aligned with a validated retrieval procedure and an appropriate timing comparison. Mechanistic, contextual, or safety evidence identifies receptor availability, signaling, circuit, cognitive, or adverse-effect constraints without directly testing the focal intervention.

These domains are intentionally non-hierarchical and are organized by the principal inference each study supports; they are not necessarily mutually exclusive in design. A systemic study may use post-reactivation dosing while serving primarily as a route- and state-dependence bridge. A rigorous clinical trial can provide high-quality evidence that a chronic regimen does not reduce cue-elicited craving while remaining indirect with respect to a retrieval-timed hypothesis. Conversely, a small local animal study can be direct for the retrieval-window question while remaining narrow in external validity. Table 1 and Figure 3 use these domains consistently throughout the manuscript.

**TABLE 1:** Evidence-domain overview of GABA_B_ reconsolidation studies and translational bridges.

Evidence domain	Model and intervention	What is demonstrated	What remains unresolved
Direct retrieval-window evidence	Morphine CPP; post-retrieval baclofen in agranular insular cortex (Sun et al., 2020)	Retrieval/no-retrieval and 6-h delayed-treatment comparisons; persistence; morphine-priming challenge; locomotor control	Local intervention in male mice within one morphine CPP paradigm; no systemic, operant, female-animal, or human test
Direct retrieval-window evidence	Morphine CPP; post-retrieval baclofen in NAcSh (Wang et al., 2020)	Retrieval/no-retrieval comparison; dose-related effect; persistence beyond 14 days; priming challenge; locomotor control	No delayed post-retrieval comparison; local intervention in male mice within one model
Extinction/expression-stage contextual evidence	Morphine or methamphetamine conditioned place preference; systemic baclofen after repeated extinction sessions; GABA_B_ PAMs after conditioning (Heinrichs et al., 2010; Voigt et al., 2011a; b)	Baclofen accelerated conditioned place preference extinction; GS39783 or CGP7930 reduced later methamphetamine conditioned place preference expression	Retrieval was embedded in extinction or was not isolated; the designs cannot distinguish retrieval-window reconsolidation from extinction consolidation, memory maintenance, or expression effects
Systemic preclinical bridge	Systemic baclofen after methamphetamine-memory reactivation (Müller and Khodamoradi, 2025)	Post-reactivation systemic effect on CPP reinstatement with age- and sleep-state dependence	Male rats; one reactivation protocol; no operant relapse test; matched retrieval and timing controls remain needed; uncertain cross-substance and clinical generalizability
Mechanistic/contextual evidence - receptor availability	VTA/NAc receptor loss, electrophysiology, and restoration (Padgett et al., 2012; Hleihil and Benke, 2024)	Addiction-state impairment of GABA_B_-GIRK signaling and behavioral effects of receptor restoration	Not retrieval-timed; does not establish memory-restabilization effects
Human pharmacology/clinical boundary	Baclofen and ASP8062 studies (Ling et al., 1998; Shoptaw et al., 2003; Kahn et al., 2009; Addolorato et al., 2007; Agabio et al., 2023; Walzer et al., 2021; Schacht et al., 2024)	Tolerability, systemic exposure, CNS pharmacodynamic effects, and mixed or negative non-timed clinical outcomes	No validated retrieval-timed design; no demonstrated reconsolidation efficacy; no direct receptor-occupancy assay
Mechanistic hypothesis	BLA PKA/ERK and NAc expression/function studies (Li et al., 2022; Zhang et al., 2022; Lan et al., 2024)	Reconsolidation-relevant pathway or regional rationale	Direct retrieval-timed GABA_B_ manipulation and pathway rescue are absent
Context/safety	Prefrontal and hippocampal evidence (Werner et al., 2020; Wu et al., 2025; Chen et al., 2026)	Contextual, abstinence-stage, plasticity, and cognitive-safety considerations	No direct addiction-memory GABA_B_ reconsolidation test

Evidence domains indicate how a study relates to the focal retrieval-timed GABA_B_, question; they are not ranks of overall methodological quality. Reported retrieval, timing, persistence, relapse-like, and behavioral-specificity controls are described directly. The published author lists of the two direct studies overlap, and this narrative search did not identify a replication by a fully non-overlapping author team.

Advancement of a direct claim requires retrieval dependence, delayed-treatment specificity, persistence after the acute drug state, relapse-like challenge, and behavioral-specificity controls. Human progression additionally requires a validated reactivation procedure and manipulation check, PK, overlap; CNS, pharmacodynamic engagement, retrieval and timing controls, and a persistent effect beyond sedation, task disengagement, and repeated cue exposure.

## 3 Reconsolidation and synaptic plasticity: essential background

### 3.1 Behavioral paradigms and minimum controls

Conditioned place preference (CPP) models a Pavlovian context-drug association. After drug-environment pairings, a brief drug-free re-exposure can retrieve the memory and, under suitable conditions, destabilize it. Immediate post-retrieval treatment, a delayed-treatment control, a no-retrieval control, long-term follow-up, reinstatement or renewal testing, and locomotor assessment help distinguish reconsolidation interference from acute expression suppression, acquisition effects, extinction, or motor impairment (Chen et al., 2021; Taujanskaitė et al., 2021).

Earlier systemic studies showed that baclofen given after repeated extinction sessions accelerated the loss of morphine or methamphetamine conditioned place preference (Heinrichs et al., 2010; Voigt et al., 2011b). These experiments are directly relevant to memory updating, but the retrieval event was embedded within prolonged or repeated extinction training. Because they did not isolate brief retrieval from extinction or include the no-retrieval and delayed-treatment comparisons needed to establish a reconsolidation window, they cannot distinguish reconsolidation interference from facilitation of extinction-memory consolidation. They are therefore treated as extinction-linked contextual evidence rather than part of the two-study direct retrieval-window core.

Self-administration models add action-outcome and cue-controlled responding and are closer to clinically relevant drug seeking. Instrumental and habit-like memories may require a stronger prediction error or a change in reinforcement contingency to destabilize than CPP memories. Therefore, a CPP effect should not be assumed to reduce compulsive or habitual seeking without confirmation in self-administration, abstinence, and relapse models (Lee et al., 2005; Everitt, 2014).

Behavioral specificity is as important as temporal specificity. A treatment that reduces locomotion, food-reinforced responding, attention, or task engagement can mimic a memory effect. Studies should therefore match the control to the behavioral demand: locomotion for CPP, inactive-lever and natural-reward responding for operant models, and attention, psychomotor, and subjective-sedation measures for human cue-reactivity tasks.

### 3.2 From glutamatergic plasticity to memory restabilization

Synaptic plasticity provides the conceptual bridge between retrieval and later behavioral persistence. N-methyl-D-aspartate receptor (NMDAR) activation permits Ca^2+^ entry that can recruit kinase signaling and alter synaptic strength. During long-term potentiation (LTP), activity-dependent delivery and redistribution of AMPA receptors can strengthen excitatory transmission; classic work demonstrated rapid AMPA-receptor delivery to spines after synaptic NMDAR activation (Shi et al., 1999). Long-term depression and memory destabilization can involve AMPA-receptor endocytosis, although the direction and function of trafficking depend on circuit and behavioral phase.

In drug-memory models, AMPA-receptor trafficking is not merely a generic plasticity marker. Amygdalar AMPA-receptor endocytosis has been implicated in disrupted reconsolidation of methamphetamine-associated contextual memory, and UCS retrieval-extinction engages related amygdala mechanisms (Yu et al., 2013; Luo et al., 2015). These findings provide context for why AMPA trafficking appears in a reconsolidation framework, but they do not establish that baclofen necessarily acts through that pathway.

GABA_B_ receptors can shape plasticity at both sides of the synapse. Presynaptic GABA_B_ receptors regulate hippocampal transmission during associative learning in behaving mice, while postsynaptic GABA_B_ receptors can inhibit dendritic L-type Ca^2+^ channels and abolish LTP at excitatory inputs onto hippocampal somatostatin interneurons (Jurado-Parras et al., 2016; Booker et al., 2018). These primary studies demonstrate that GABA_B_ signaling can alter plasticity, but neither is a drug-memory reconsolidation experiment.

Restabilization also recruits intracellular and transcriptional processes, including PKA, extracellular signal-regulated kinase (ERK), cAMP response element-binding protein (CREB), immediate-early genes, and protein synthesis. In heroin-memory models, post-retrieval inhibition of basolateral-amygdala PKA or ERK reduced later seeking under retrieval and timing controls (Li et al., 2022; Zhang et al., 2022). These studies establish pathway requirements in the basolateral amygdala, not direct GABA_B_-to-PKA/ERK causality.

### 3.3 Established receptor actions versus candidate convergence

The most defensible mechanistic chain begins with established receptor actions: reduced presynaptic Ca^2+^ entry and glutamate release, increased postsynaptic GIRK conductance, and reduced AC-cAMP-PKA signaling. Candidate convergence begins where these actions are proposed to lower NMDAR-related excitation, kinase activation, CREB-dependent transcription, protein synthesis, AMPA-receptor trafficking, or synaptic remodeling during restabilization. Figure 2 marks this boundary by distinguishing canonical signaling from indirect or context-dependent correspondence.

Accordingly, changes in ERK, CREB, brain-derived neurotrophic factor, immediate-early genes, or receptor trafficking should be interpreted as pathway hypotheses unless a retrieval-timed GABA_B_ experiment measures the node and demonstrates rescue or blockade. This distinction shortens the mechanistic argument while preserving testable predictions.

## 4 GABA_B_ receptor pharmacology relevant to the retrieval window

### 4.1 Synaptic localization and canonical signaling

Functional GABA_B_ receptors are obligate GB1-GB2 heterodimers. GB1 contributes orthosteric ligand recognition and trafficking partnership, whereas GB2 is the principal G-protein-coupling subunit. Structural studies have resolved agonist-dependent rearrangements and Gi coupling, supporting rational ligand design without implying reconsolidation efficacy (Shaye et al., 2020; Shen et al., 2021).

At presynaptic terminals, Gβγ-mediated inhibition of voltage-gated Ca^2+^ channels reduces transmitter release. At postsynaptic sites, Gβγ activates GIRK channels and promotes hyperpolarization. In parallel, Gαi/o inhibits AC, lowers cAMP, and reduces PKA signaling. Figure 1 places the GB1-GB2 heterodimer in this synaptic context and retains only the subunit labels needed to orient orthosteric ligand recognition and G-protein coupling.

The timing of these effects matters. When a drug is given after retrieval, reduced excitation is most plausibly interpreted as constraining restabilization-related activity rather than preventing the retrieval event itself. However, inadequate task engagement, sedation, or acute motivational suppression can still reduce the measured response; post-retrieval timing does not remove the need for specificity controls.

### 4.2 Auxiliary regulation, receptor availability, and candidate pathways

Auxiliary potassium channel tetramerization domain-containing (KCTD) proteins can alter agonist potency, signaling kinetics, and desensitization. KCTD-dependent uncoupling of Gβγ from effector channels explains why sustained receptor occupancy need not produce a sustained inhibitory current (Schwenk et al., 2010; Seddik et al., 2012; Turecek et al., 2014; Zheng et al., 2019). These mechanisms are relevant to candidate selection but are not evidence of drug-memory reconsolidation.

Receptor availability may also constrain pharmacological responsiveness. Protein phosphatase 2A (PP2A) can interact with GABA_B_ receptors and alter signaling, while phosphorylation and recycling at serine 783 influence surface expression (Li et al., 2020; Hleihil et al., 2022). In methamphetamine-related models, reduced ventral tegmental area (VTA) GABA_B_ receptor signaling and receptor restoration are associated with changes in sensitization and drug seeking (Padgett et al., 2012; Hleihil and Benke, 2024). These findings support an availability hypothesis but do not establish a retrieval-window effect.

The candidate pathway model is therefore staged: receptor localization and canonical coupling are established; auxiliary regulation and receptor availability influence the size and duration of the inhibitory response; and convergence with PKA/ERK/CREB, protein synthesis, or AMPA-receptor trafficking remains to be demonstrated in retrieval-timed experiments. Figure 2 depicts this staged interpretation rather than a single proven linear pathway.

### 4.3 Orthosteric agonism, positive allosteric modulation, and specificity

Baclofen provides a clinically available orthosteric probe but can produce somnolence, dizziness, motor effects, and cognitive or attentional impairment (Agabio et al., 2023). These effects narrow the separation between pharmacological action and nonspecific response suppression. Candidate PAMs may offer a wider functional window by amplifying endogenous GABA signaling, but intrinsic activity, receptor reserve, brain penetration, native subunit composition, and tolerability vary among compounds (Vengeliene et al., 2018; Maccioni and Colombo, 2024; Wydra et al., 2024).

An earlier primary study found that acute GS39783 or CGP7930, administered after methamphetamine conditioning, reduced subsequent expression of established conditioned place preference (Voigt et al., 2011a). Because treatment was not coupled to an isolated retrieval event, the finding supports GABA_B_-PAM effects on memory maintenance or cue salience but does not establish retrieval-window reconsolidation.

Preclinical baclofen-versus-PAM comparisons report reductions in alcohol- or cocaine-related behaviors with a potentially wider separation from locomotor or food-response suppression for some PAMs (Vengeliene et al., 2018; Maccioni and Colombo, 2024; Wydra et al., 2024). The relevant lesson is the paired efficacy-specificity assessment, not a class-wide assumption that all PAMs are safer or more effective.

Human pharmacology should be interpreted in distinct layers. Plasma concentrations and pharmacokinetics establish systemic exposure and window coverage. EEG, PSG, GH, or another validated compound-specific readout may indicate CNS pharmacodynamic engagement. Cognitive, psychomotor, and sedation measures primarily assess safety and interpretive confounding, whereas cue reactivity and substance-use outcomes assess memory-relevant or clinical effects. None of these measures should be described as direct receptor-specific target engagement unless a validated assay demonstrates receptor occupancy or an equivalently specific interaction. Human evidence is considered as a distinct domain in Section 5.4.

## 5 Evidence domains: demonstrated effects and translational bridges

Table 1 and Figure 3 organize the literature by evidence domain. Direct retrieval-window studies are kept separate from systemic preclinical bridges, human pharmacology or clinical-boundary studies, and mechanistic, contextual, or safety evidence. This structure is non-comparative: a rigorous mechanistic or clinical study may be highly informative while still not answering the focal retrieval-timed question.

### 5.1 Direct retrieval-window evidence: agranular insular cortex and nucleus accumbens shell

The agranular insular cortex (AI) study reported the broader retrieval-window control set among the two local experiments. Immediate post-retrieval microinjection of baclofen into bilateral AI disrupted morphine conditioned place preference, whereas a 6 h delayed intervention and administration without retrieval were ineffective. The reported effect persisted, resisted morphine-primed reinstatement, occurred without locomotor impairment, and was accompanied by reduced ΔFosB expression (Sun et al., 2020).

This result supports a specific conclusion: under the tested retrieval conditions, local AI GABA_B_ activation can interfere with morphine CPP restabilization. It does not establish substance-general efficacy or show that every insular contribution to craving reflects reconsolidation. The broader insular literature supports roles in interoception and context-evoked drug craving, but expression-stage functions and durable post-retrieval modification should remain distinct (Naqvi and Bechara, 2010; Contreras et al., 2012).

The nucleus accumbens shell (NAcSh) study provides a second direct post-retrieval result. Following morphine-memory retrieval, intra-NAcSh baclofen reduced CPP in a dose-related manner; no-retrieval and locomotor controls were negative, the effect persisted for more than 14 days, and morphine priming did not restore CPP (Wang et al., 2020). Because no delayed post-retrieval comparison was reported, the study demonstrates retrieval dependence and durability but leaves time-window specificity unresolved.

Taken together, the two studies form the direct behavioral core of the field. The AI study reported retrieval/no-retrieval and immediate/delayed-treatment comparisons; the nucleus accumbens shell study reported retrieval/no-retrieval contrasts but no delayed-treatment comparison. Both used local baclofen in male mice with morphine conditioned place preference, so neither establishes systemic efficacy, operant relapse prevention, sex generalizability, or clinical benefit. Their published author lists overlap, and this narrative search did not identify a replication by a fully non-overlapping author team. The highest-priority replication is a matched design across the agranular insular cortex and nucleus accumbens shell with immediate, delayed, and no-retrieval conditions, post-acute persistence and relapse-like tests, and non-drug reward controls.

### 5.2 Systemic preclinical bridge

Systemic post-reactivation dosing is an essential bridge beyond local microinjection. In adolescent and adult male rats studied under control and rapid-eye-movement-sleep-deprived conditions, immediate post-reactivation intraperitoneal baclofen reduced methamphetamine conditioned place preference reinstatement, with effects varying by age and sleep state (Müller and Khodamoradi, 2025). The study directly tested a post-reactivation systemic regimen and provides route-level proof of principle, but it remains substance-, species-, paradigm-, sex-, age-, and state-dependent and does not remove the need for matched retrieval/no-retrieval and immediate/delayed comparisons.

A decisive systemic program should extend beyond CPP to self-administration and relapse models, align brain exposure with the post-retrieval interval, and include inactive-lever, natural-reward, locomotor, and cognitive controls. Without such controls, reduced seeking can remain ambiguous between memory modification, altered motivation, and nonspecific suppression.

### 5.3 Mechanistic, contextual, and safety evidence

The basolateral amygdala (BLA) is a strong mechanistic candidate because post-retrieval PKA and ERK inhibition reduces heroin seeking under timing and retrieval controls (Li et al., 2022; Zhang et al., 2022). However, direct retrieval-timed BLA GABA_B_ manipulation has not been reported. A pathway claim will require local GABA_B_ intervention, measurement of the proposed signaling node, and rescue or blockade showing that the node mediates the behavioral effect.

VTA evidence supports an addiction-state receptor-availability problem rather than reconsolidation. Methamphetamine exposure depresses GABA_B_-GIRK signaling, and restoring receptor expression can reduce sensitization and drug seeking (Padgett et al., 2012; Hleihil and Benke, 2024). These results may explain why increasing agonist dose is insufficient when receptor availability is reduced, but they do not show that VTA manipulation changes post-retrieval restabilization.

Nucleus-accumbens expression data similarly provide mechanistic plausibility rather than a direct memory-window test. GABA_B_ receptor function and messenger RNA expression are altered in cocaine-exposed rats, but messenger RNA does not establish surface receptor abundance, coupling efficiency, or causal direction (Lan et al., 2024).

Prefrontal and hippocampal evidence primarily defines context and safety boundaries. Prolonged-abstinence hippocampal adaptations contribute to cocaine seeking, while non-addiction studies show that GABA_B_ activation or blockade can affect hippocampal plasticity and memory in opposite directions depending on baseline state (Werner et al., 2020; Wu et al., 2025; Chen et al., 2026). Systemic trials should therefore monitor attention, executive function, contextual memory, sleep, and cognitive vulnerability rather than assuming that stronger inhibition is uniformly beneficial.

### 5.4 Human pharmacology and clinical-boundary evidence

Published human baclofen studies were not designed around a retrieval-defined reconsolidation window. A preliminary cocaine anti-craving report was followed by a randomized trial in which baclofen produced a later increase in cocaine-negative urine samples in exploratory analyses without a parallel reduction in subjective craving (Ling et al., 1998; Shoptaw et al., 2003). A larger multicenter trial then failed to improve abstinence initiation, retention, or overall cocaine use (Kahn et al., 2009). These outcomes should not be treated as interchangeable indicators of efficacy.

Alcohol-use-disorder findings are also population- and endpoint-dependent. In patients with alcohol dependence and liver cirrhosis, baclofen increased continuous abstinence in one randomized trial, whereas a broader Cochrane review found modest and heterogeneous effects across outcomes and no consistent class-wide dose-response pattern (Addolorato et al., 2007; Agabio et al., 2023). This literature defines a possible clinical niche and safety experience, not retrieval-dependent memory modification.

The GABA_B_ PAM ASP8062 provides a useful human-pharmacology and clinical-boundary example. A phase 1 study in healthy participants showed measurable pharmacokinetic, sleep, EEG, and endocrine effects consistent with CNS pharmacodynamic activity (Walzer et al., 2021). In a later multisite alcohol-use-disorder trial, ASP8062 was well tolerated but did not reduce cue-elicited craving or naturalistic drinking at the tested regimen (Schacht et al., 2024). Neither study used retrieval-timed dosing, and neither established direct GABA_B_ receptor occupancy.

Human evidence therefore answers several questions separately: whether a compound can be administered safely, whether systemic exposure covers the intended interval, whether a CNS pharmacodynamic response is measurable, and whether a defined patient outcome improves. None of these automatically demonstrates reconsolidation. A future trial must add a validated reactivation procedure, a retrieval manipulation check, an appropriate timing comparison, and post-acute persistence testing while retaining exposure, CNS pharmacodynamic, sedation, and clinical outcome measures.

### 5.5 Evidence-domain summary

Figure 3 displays the evidence domains without implying anatomical direction or comparability among them. Table 1 provides the corresponding study-level interpretation, including reported controls and unresolved questions. The overview makes the two local morphine CPP studies visible as the direct core while preventing mechanistic and human studies from being misread as equivalent retrieval-window evidence.

## 6 Discussion

### 6.1 A bounded restabilization-constraining model

The most parsimonious model is not memory erasure. When a defined drug memory has been retrieved and destabilized, GABA_B_ activation may lower presynaptic glutamatergic drive, postsynaptic excitability, and AC-cAMP-PKA support below the threshold required for full restabilization. The AI timing control and the retrieval-dependence controls in AI and NAcSh are consistent with this interpretation (Sun et al., 2020; Wang et al., 2020).

The model does not require one universal downstream molecule. PKA/CREB, ERK-related signaling, protein synthesis, AMPA-receptor trafficking, receptor availability, or other plasticity processes may dominate in different circuits and memory phenotypes. These nodes are hypotheses until a retrieval-timed GABA_B_ experiment measures and causally tests them.

The model is also state dependent. Receptor availability, auxiliary-subunit composition, sleep, age, abstinence stage, memory strength, and drug class can change the response to the same ligand. A null effect can therefore reflect failure to open the window, inadequate exposure, absent or insufficient CNS pharmacodynamic engagement, sedation or task disengagement, assay insensitivity, or true pharmacological failure; study design must discriminate among these possibilities.

### 6.2 Boundary conditions and limits of the present evidence

The direct evidence is narrow: two local baclofen studies in male mice using morphine conditioned place preference. The published author lists overlap, and this narrative search did not identify a replication by a fully non-overlapping author team. Because literature identification was narrative rather than systematic, that absence statement is limited to the sources reviewed and is not proof of exhaustive nonexistence. Conditioned place preference captures a context-reward association but not the full social, interoceptive, affective, executive, and habit components of human relapse. Extension to self-administration and relapse models is necessary before broad treatment claims.

This review has corresponding methodological limitations. It was not preregistered, did not use duplicate screening, did not enumerate an exhaustive study flow, and did not apply a formal risk-of-bias instrument. The evidence-domain framework is therefore an interpretive device for separating claims, not a quantitative estimate of literature completeness or comparative methodological quality.

Retrieval itself is a nonlinear gate. Weak reactivation may fail to destabilize the target memory, whereas prolonged nonreinforcement may engage extinction. Prediction error should be manipulated or measured prospectively, and the target association—discrete cue, context, interoceptive state, action-outcome memory, or habit-like response—should be specified rather than assumed (Exton-McGuinness et al., 2015; Kuijer et al., 2020).

Drug class and patient state also limit generalization. Opioid, psychostimulant, alcohol, and nicotine memories differ in cue structure, withdrawal-related bodily states, legal availability, and repeated environmental exposure. Cross-substance development should therefore proceed by memory phenotype and retrieval procedure, not receptor pharmacology alone (Barak and Goltseker, 2021).

Sex remains an unresolved biological variable in the direct evidence. Future preclinical work should include female animals and report hormonal state where relevant, while clinical studies should examine whether sex, sleep, stress responsivity, pharmacokinetics, CNS pharmacodynamic responses, or adverse effects modify retrieval or treatment response. Findings from restricted samples should not be assumed to generalize to all patients.

### 6.3 Candidate selection and behavioral specificity

Baclofen is the most practical repurposing probe, not an optimized therapy. Its value is the availability of human dosing and safety information; its limitation is the overlap between potentially useful inhibition and somnolence, motor, cognitive, or attentional effects. An early study should use a dose selected to provide adequate exposure and measurable CNS pharmacodynamic activity rather than simply the maximum tolerated dose, and should prespecify psychomotor and cognitive controls.

PAMs are rational second-stage candidates when they demonstrate brain exposure, activity-dependent receptor modulation, and a reproducible separation from motor or natural-reward suppression. The negative ASP8062 trial prevents a class-wide efficacy assumption and makes dose, regimen, exposure, CNS pharmacodynamic engagement, retrieval timing, and endpoint selection explicit development variables (Schacht et al., 2024).

Receptor-availability strategies, including PP2A-related trafficking modulation, are longer-term options. They should advance only after feasible delivery is established and after experiments distinguish effects on baseline seeking from effects on post-retrieval restabilization.

### 6.4 Human proof-of-concept assessment

The first clinical study should be framed as a mechanistic proof of concept rather than treatment for all substance use disorders. A suitable initial population would be abstinent or clinically stable participants with reproducible cue reactivity, capacity to consent, and low acute sedation or relapse risk. The core question is whether active treatment after standardized memory reactivation produces a persistent, retrieval- and timing-dependent change in memory-related responding after acute drug effects have resolved, beyond sedation, task disengagement, and repeated cue exposure.

The reactivation procedure should specify cue modality, duration, prediction error, personalization, and target memory. A mechanistic trial should include reactivation plus active drug and reactivation plus placebo; a matched neutral/no-reactivation plus active-drug arm is needed to test retrieval dependence, and a reactivation plus delayed or off-window active-drug arm should be included when feasible to test timing dependence. Manipulation checks can confirm target-memory activation and the planned prediction error, but they cannot by themselves prove destabilization; the pattern across retrieval and timing control arms carries the mechanistic inference. Video, odor, imagery, context, or virtual-reality cues can be selected according to the dominant memory phenotype. Because cue exposure may transiently increase craving, studies require supervision, stopping thresholds, observation, rescue procedures, and post-session relapse-support contact. All randomized arms should receive the same standard clinical support and relapse-risk management.

Pharmacokinetic/pharmacodynamic (PK/PD) alignment is essential. Plasma drug concentrations and pharmacokinetics should establish systemic exposure during the intended post-retrieval interval. EEG, PSG, GH, or another validated compound-specific measure may provide evidence of CNS pharmacodynamic engagement; a receptor-specific target-engagement claim requires a validated direct assay. Sedation, attention, cognition, and psychomotor performance should be analyzed as specificity and safety measures rather than pooled with exposure or CNS pharmacodynamic measures (Walzer et al., 2021).

Human outcomes should be layered. Retrieval manipulation checks should document activation of the intended memory and the planned prediction error. Proximal memory-relevant outcomes may include cue-elicited craving, attention bias, and physiological arousal, but the primary mechanistic outcome should be reassessed after acute drug effects have resolved. Intermediate and clinical outcomes include lapse, urine toxicology, days of use, and time to relapse. Longitudinal follow-up should test whether a persistent early change predicts clinically meaningful benefit. A change in craving, an increase in drug-negative samples, and successful abstinence initiation are not interchangeable endpoints.

Sedation is both a safety issue and an interpretive confound. Ratings of somnolence, motor coordination, attention, memory, driving risk, and falls should be prespecified. If reduced cue reactivity is fully explained by sedation or task disengagement, the pharmacological strategy has not demonstrated a memory-specific effect.

### 6.5 Go/no-go and falsification criteria

Clinical progression requires four linked demonstrations: a reproducible reactivation procedure with a manipulation check, PK coverage during the intended interval, measurable CNS pharmacodynamic engagement without prohibitive confounding, and a persistent retrieval- and timing-dependent memory-relevant effect after the acute drug state. Figure 4 provides a high-level overview of the development stages; the mechanistic control structure and interpretive requirements are detailed in Section 6.4 and Table 2, while Table 3 summarizes candidate development.

**TABLE 2:** Human mechanistic proof-of-concept domains, measures, and go/no-go rules.

Assessment domain	Measures or design features	Interpretive purpose	Risk control/go-no-go rule
Patient selection	Substance class, memory phenotype, abstinence stage, sex, age, sleep, cognition, baseline cue reactivity, psychiatric stability, and sedation vulnerability	Define a clinically stable population and reduce uninterpretable heterogeneity	Exclude unstable withdrawal, impaired consent, severe sedation risk, or high acute relapse risk
Reactivation and manipulation check	Cue modality, duration, prediction error, personalization, target-memory activation, and immediate retrieval response	Document target-memory activation and planned prediction error; this does not by itself prove destabilization	Supervision, stopping thresholds, observation, rescue plan, and prespecified evidence of adequate reactivation
Randomized mechanistic control structure	Reactivation + active drug; reactivation + placebo; matched neutral/no-reactivation + active drug; optional delayed/off-window active drug	Test whether any persistent effect depends on both retrieval and treatment timing	Do not infer reconsolidation from a single active-treatment arm, a manipulation check alone, or repeated cue exposure; provide the same standard clinical support across arms
Systemic exposure/PK coverage	Dose, concentration-time profile, time to maximum concentration, half-life, and overlap with the intended post-retrieval interval	Establish that pharmacological exposure covers the proposed window	Do not interpret a negative study as target failure when exposure is inadequate
CNS pharmacodynamic engagement	EEG/PSG, GH, or another validated compound-specific CNS pharmacodynamic readout; receptor-specific assay when available	Show a measurable CNS pharmacodynamic response without equating nonspecific biomarkers with receptor occupancy	A receptor-specific target-engagement claim requires a validated direct assay
Specificity and confound control	Sedation, attention, cognition, psychomotor performance, task engagement, expectancy, and blinding integrity	Separate memory modulation from acute suppression or disengagement	A reduced cue response fully explained by sedation or poor task engagement is not a memory-specific effect
Memory-relevant and clinical outcomes	Post-acute cue reactivity, craving, attention bias, physiological arousal, urine toxicology, lapse, days of use, time to relapse, adverse events, and follow-up	Test persistence after acute drug effects and link early mechanistic effects to clinically meaningful benefit	Prespecified relapse-support plan and confirmation that the primary mechanistic effect persists beyond the acute drug state

Early trials should distinguish systemic exposure, CNS, pharmacodynamic engagement, retrieval manipulation, specificity controls, and memory-relevant outcomes. Direct receptor-specific target engagement should be claimed only when supported by a validated assay.

**TABLE 3:** Staged development roadmap for retrieval-timed GABA_B_ intervention.

Component	Development role	Current rationale	Next decision
Baclofen	Repurposing proof-of-concept probe	Clinically available; supported by two local morphine CPP studies and a systemic post-reactivation methamphetamine study, but human efficacy is heterogeneous and sedation-sensitive	Align retrieval, PK coverage, CNS pharmacodynamic engagement, post-acute outcomes, and specificity controls before treatment claims
GABA_B_ PAMs	Second-stage pharmacological optimization	Activity-dependent amplification and preclinical efficacy-specificity separation; human CNS pharmacodynamics are measurable, but ASP8062 did not reduce alcohol cue craving or drinking at the tested regimen	Demonstrate dose, window coverage, CNS pharmacodynamics, and a persistent effect beyond sedation; do not assume class-wide efficacy
PP2A/Ser783 and receptor trafficking	Longer-term receptor-availability strategy	Links receptor phosphorylation, recycling, and inhibitory tone to addiction-state responsiveness	Establish feasible delivery and test whether restoration changes post-retrieval restabilization rather than baseline seeking
Standardized CS or UCS retrieval	Behavioral opening operation	Retrieval-dependent updating can reduce relapse-related responses, but effects vary with cue type, prediction error, timing, and relapse metric	Prespecify target memory, retrieval strength, interval, manipulation check, and safety procedures before interpreting pharmacological failure
Mechanistic control architecture	Causal test of retrieval and timing dependence	The same active drug can affect craving, arousal, attention, or behavior outside reconsolidation	Compare reactivation + active, reactivation + placebo, matched neutral/no-reactivation + active, and, when feasible, reactivation + delayed/off-window active; provide the same standard clinical support across arms and reassess after acute effects resolve

The roadmap separates immediate mechanistic proof-of-concept testing from longer-term pharmacological optimization. It does not imply that any candidate is already proven for relapse prevention.

A study that is negative despite verified reactivation, adequate exposure, measurable CNS pharmacodynamic engagement, appropriate retrieval and timing controls, and behavioral-specificity measures would materially narrow or falsify the GABA_B_-window hypothesis for that population and regimen. A negative study missing one of these elements remains procedurally ambiguous and should trigger targeted optimization rather than an efficacy claim.

The same standards apply preclinically. Advancement requires matched immediate, delayed, and no-retrieval conditions, long-term and relapse-like testing, and behavioral controls appropriate to the task. Repeated null effects under these conditions would be more informative than adding mechanistic correlates to an inadequately controlled paradigm.

## 7 Conclusion

Direct retrieval-window evidence for GABA_B_ modulation remains limited to two local baclofen studies in male mice using morphine conditioned place preference. The agranular insular cortex study reported retrieval/no-retrieval and immediate/delayed-treatment comparisons; the nucleus accumbens shell study was retrieval-dependent and durable but did not report a delayed-treatment comparison. Earlier repeated-extinction baclofen studies and post-conditioning PAM studies provide contextual support but do not isolate a retrieval window. The published author lists of the two direct studies overlap, and a replication by a fully non-overlapping author team was not identified in this narrative search. Systemic post-reactivation baclofen provides a preclinical bridge, while receptor-availability studies and human baclofen or ASP8062 data define mechanism, exposure, CNS pharmacodynamic effects, and clinical boundaries rather than retrieval-timed efficacy.

The field should prioritize independent replication of the local findings, followed by systemic retrieval-timed studies in self-administration and relapse models, direct pathway tests in candidate circuits, matched comparisons of baclofen and PAMs, and early clinical experiments that verify memory reactivation, pharmacokinetic coverage, CNS pharmacodynamic engagement, retrieval and timing dependence, and post-acute persistence beyond sedation or task disengagement. The current evidence supports a falsifiable neuropharmacological development program, not memory deletion or a universal anti-addiction therapy.
